# Modulation of Saliva Microbiota through Prebiotic Intervention in HIV-Infected Individuals

**DOI:** 10.3390/nu11061346

**Published:** 2019-06-14

**Authors:** Nuria Jiménez-Hernández, Sergio Serrano-Villar, Alba Domingo, Xavier Pons, Alejandro Artacho, Vicente Estrada, Andrés Moya, María José Gosalbes

**Affiliations:** 1 Área de Genómica y Salud, Fundación para el Fomento de la Investigación Sanitaria y Biomédica de la Comunitat Valenciana (FISABIO), 46020 Valencia, Spain; jimenez_nurher@gva.es (N.J.-H.); aldope@alumni.uv.es (A.D.); pons_javtam@gva.es (X.P.); artacho_ale@gva.es (A.A.); andres.moya@uv.es (A.M.); 2 CIBER en Epidemiología y Salud Pública, 28029 Madrid, Spain; 3 Departamento de Enfermedades Infecciosas, Hospital Universitario Ramón y Cajal, 28034 Madrid, Spain; serranovillar@gmail.com; 4 Unidad de Enfermedades Infecciosas/Medicina Interna, Hospital Clínico San Carlos-IdiSSC, Universidad Complutense, 28040 Madrid, Spain; vesda001@gmail.com; 5 Instituto de Biología Integrativa de Sistemas, Universidad de Valencia y CSIC, 46980 Paterna, Valencia, Spain

**Keywords:** prebiotic intervention, saliva, microbiota, HIV, bacteria co-occurrence, gut

## Abstract

Human immunodeficiency virus (HIV) infection is characterized by an early depletion of the mucosal associated T helper (CD4+) cells that impair the host immunity and impact the oral and gut microbiomes. Although, the HIV-associated gut microbiota was studied in depth, few works addressed the dysbiosis of oral microbiota in HIV infection and, to our knowledge, no studies on intervention with prebiotics were performed. We studied the effect of a six-week-long prebiotic administration on the salivary microbiota in HIV patients and healthy subjects. Also, the co-occurrence of saliva microorganisms in the fecal bacteria community was explored. We assessed salivary and feces microbiota composition using deep 16S ribosomal RNA (rRNA) gene sequencing with Illumina methodology. At baseline, the different groups shared the same most abundant genera, but the HIV status had an impact on the saliva microbiota composition and diversity parameters. After the intervention with prebiotics, we found a drastic decrease in alpha diversity parameters, as well as a change of beta diversity, without a clear directionality toward a healthy microbiota. Interestingly, we found a differential response to the prebiotics, depending on the initial microbiota. On the basis of 100% identity clustering, we detected saliva sequences in the feces datasets, suggesting a drag of microorganisms from the upper to the lower gastrointestinal tract.

## 1. Introduction

The oral microbiome was established along evolution-specific symbiotic interactions with the host, such that the microbiota of the oral cavity plays an important role in pathogen colonization resistance and local immune system modulation, as well as in the entero-salivary nitrate reduction cycle [1]. In the last few years, due to a decrease in the effectiveness of conventional antimicrobial therapy, new strategies such as the application of probiotics or prebiotics were investigated to prevent different oral diseases [2,3,4,5,6,7]. However, different studies suggested that probiotic strains, widely used to provide health benefits in the gut, are not as efficient in the care of oral health [3,4]. Recently, oral *Streptococcus* strains, isolated from caries-free cavities, were proposed as effective probiotics in caries prevention due to their inhibitory action of the major oral pathogens and their buffering of acidic pH [5,7]. However, the use of prebiotics in oral health is in its infancy. Recently, Slomka et al. [8,9] evaluated different potential oral prebiotics and two compounds (beta-methyl-d-galactoside and *N*-acetyl-d-mannosamine) were identified as substrates with a stimulatory effect on beneficial oral bacteria growth and the subsequent suppression of pathogens. In caries management, compounds such as urea or arginine are considered to be prebiotics since their metabolism by oral bacteria produces alkali that increase the pH, inhibiting acidogenic and aciduric pathogens. Other studies indicated that dietary nitrate increases the abundance of *Neisseria* and *Rothia* species associated with oral health [10]. Changes in acid–alkali balance lead to an oral dysbiotic microbiota. High pH is associated with a high abundance of proteolytic bacteria, promoting gingival or periodontal inflammation. On the other hand, an acidic environment correlates with a saccharolytic microbiota, resulting in caries. Thus, nutritional strategies should be individually tailored to obtain higher benefits.

The explosion of the microbiome field shook our understanding of the pathogenesis of HIV, in which the participation of bacteria in the course of the disease was far from suspected. There is emerging consensus, however, that disturbances in the gut microbial ecology during HIV infection correlate with chronic immune defects and inflammation [11]. Some studies assessing dietary supplementation with different products, including prebiotics [12,13,14], probiotics [15], bovine colostrum [16], or a combination of different ingredients [17], collectively suggested that these strategies may exert some systemic beneficial immunological effects introducing changes in the HIV-associated gut microbiome. Studies on HIV-associated oral microbiota [18,19,20,21,22] relying on culture-dependent and -independent techniques, indicated an effect of both HIV infection and antiretroviral treatment on the bacterial community composition. However, the scope of HIV-associated dysbiosis in the oral cavity, the associations with systemic predictors of clinical progression, and the interplay between oral and gut microbiome remain poorly defined. Moreover, no studies were done on the role of prebiotic application aimed at manipulating the HIV-associated oral microbiome.

In a previous work [14], we studied the effect of a mixture of prebiotics (short-chain galacto-oligosaccharides, long-chain fructo-oligosaccharides, and glutamine) on the HIV-associated gut microbiota. During this nutritional intervention, we collected fecal and salivary samples from HIV-infected patients. Thus, in the present study, we characterized, for the first time, the compositional changes associated with prebiotic intervention on salivary microbiota in HIV-infected individuals. Furthermore, we studied the interplay between oral and gut microbiota determining the bacterial co-occurrences in both habitats.

## 2. Material and Methods

### 2.1. Subjects and Sample Collection

We conducted the study with participants belonging to a previously described cohort [14]. Briefly, we recruited 95 individuals from two University hospitals in Madrid, Spain (University Hospital Clínico San Carlos and University Hospital Ramón y Cajal) with 35 of them being ineligible (Figure S1, Supplementary Materials). Exclusion criteria were the concomitant use of medications, the use of systemic antibiotics, probiotics, or prebiotics during the previous three months, and any acute or chronic condition other than chronic HIV infection, including gastrointestinal symptoms or co-infections with hepatitis B or C viruses. The inclusion criteria were serologically documented HIV infection, age 18 years or older, and, for antiretroviral therapy (ART)-treated patients, at least two years under ART-mediated HIV RNA suppression. The controls were healthy non-HIV-infected volunteers. From the 60 individuals included in the cohort, we obtained salivary samples for 53 individuals at baseline that were grouped in viremic ART-untreated (VU, *n =* 12), immunological ART responders (IR, *n =* 18), immunological ART non-responders (INR, *n* = 9), and HIV-uninfected individuals as controls (*n =* 14) (Table 1). A total of 32 individuals completed the six-week prebiotic intervention. The prebiotics (20 g) consisted of a mixture of short-chain galacto-oligosaccharides (5 g), long-chain fructo-oligosaccharides (10 g), and glutamine (5 g), which is an energy source for enterocytes. The prebiotics supplier was Nutricia (Nutricia, S.R.L., Dirección Ctra. de Andalucía, Km. 25.6, 28340, Madrid). The study individuals completed a dietary survey and no differences were detected among the groups in dietary habits [14]. The safety of the intervention was evaluated in the previous work [14]. Clinical parameters, such as plasma metabolic profile, T-cell markers, thymic function, endothelial function, bacterial translocation, inflammation, and thrombosis, were measured in previous work [14].

Unstimulated salivary samples (approximately 2 mL) were collected in sterile tubes at midday (after 5 h of personal oral hygiene) during the visit of the patients to the HIV unit in the hospitals at baseline and after six weeks. The salivary samples were kept at −80 °C until use. Collection of fecal samples was described in Reference [14].

All subjects gave their informed consent for inclusion before they participated in the study. The study was conducted in accordance with the Declaration of Helsinki, and the protocol was approved by the Ethics Committee of the Public Health Department and the Center for Public Health Research (DGSP-CSISP), Valencia, Spain and by the Ethics Committees of both recruiting institutions (University Hospital Clínico San Carlos, ceic.hcsc@salud.madrid.org and University Hospital Ramón y Cajal, ceic.hrc@salud.madrid.org) (approval number 11/284).

### 2.2. Bacterial DNA Extraction and Sequencing

Total DNA was extracted in the robotic workstation MagNA Pure LC Instrument (Roche) using the MagNA Pure LC DNA isolation kit III (Bacteria, Fungi) (Roche). Previously, salivary samples (300 μL of saliva) were centrifuged at maximum speed at 4 °C for 5 min to pellet the cells. Bacterial pellets were resuspended in lysis buffer (MagNA Pure LC DNA isolation kit III) and treated for one hour at 37 °C with an enzymatic cocktail (lysozyme 25 mg/mL, lysostaphin 1.25 mg/mL, mutanolysin 625 U/mL). Then, the samples were treated for 15 min at 65 °C with proteinase K according to the instructions of MagNA Pure LC DNA isolation kit III. Total DNA was quantified with a Qubit Fluorometer (ThermoFisher) and its integrity was verified by standard agarose gel electrophoresis.

We amplified the V3–V4 region of the 16S rRNA gene from total DNA obtained from salivary and fecal samples. The amplicon libraries were constructed following Illumina instructions. The libraries were quantified with a Qubit Fluorometer (ThermoFisher) and sequenced using the Kit v3 (2 × 230 cycles) in a MiSeq platform (Illumina) at FISABIO-Salud Pública. All the sequences were deposited in the European Bioinformatics Institute (EBI) database under the number PRJEB25569.

### 2.3. Analysis of 16S rRNA Gene Amplicons

A quality assessment of the raw reads was performed using the prinseq-lite program [23] applying the following parameters: min_length: 50, trim_qual_right: 30, trim_qual_type: mean, and trim_qual_window: 20. R1 and R2 reads from Illumina sequencing were joined using the *FLASH* program applying default parameters [24]. The 16S rDNA sequences were processed using the QIIME pipeline (v2-2017.12). Operational taxonomic units (OTUs) were obtained at 97% sequence similarity by clustering with the USEARCH software in the QIIME pipeline. Taxonomic information of the 16S rDNA sequences was obtained using the Ribosomal Database Project (RDP) naïve Bayesian classifier algorithm with the GreenGenes database (version gg_13_5) in the QIIME pipeline. The annotation was accepted when the bootstrap confidence estimation value was over 0.8, and the assignation was stopped at the last well-identified phylogenetic level. To obtain weighted and unweighted UniFrac distance matrices, we firstly generated a phylogenetic tree and then applied the core-metrics-phylogenetic method using the QIIME pipeline.

### 2.4. Quantitative PCR

To quantify the bacterial load in salivary samples, we performed real-time PCR using LightCycler 480 instrument (Roche) and SYBR Premix ExTaq (Tli RNaseH Plus) (Takara Bio Europe). Amplification reactions were performed in 20 μL final volume with 1 μL of total DNA purified from salivary samples as a template and using the universal bacterial primers 16S-U515F and 16S-U789R. All the reactions were made in duplicate. The bacterial load was calculated by comparison with the crossing point (cp) values obtained from a standard curve. The standard curve was prepared using serial dilutions of DNA extracted from a bacterial suspension that contained 10 million cells. The bacteria were quantified and sorted by flow cytometry as described previously [25]. The results were expressed as number of cells per mL of saliva.

### 2.5. Statistical Analysis

The alpha diversity was determined at the OTU level using the vegan library (function reny and diversity) from the R package version 3.2.0 [26] that gives, as default, observed OTUs, Shannon diversity index, Simpson index of diversity (1-D), Chao1 richness estimator, and an Abundance-based Coverage Estimator (ACE). To analyze beta diversity, we applied principal coordinate analysis (PCoA) based on weighted and unweighted UniFrac distances. Box plots, sample clustering, canonical correspondence analysis (CCA), and heatmaps were generated with in-house R scripts. To statistically assess the effect of the environmental factors on the bacterial composition, a multivariate analysis of variance based on dissimilarity test (ADONIS) was applied using the vegan library from the R package (adonis function). To evaluate differences between groups in continuous variables, we used the Kruskal–Wallis test. The pairwise comparisons of continuous variables were analyzed using the Wilcoxon rank-sum test. The alpha values for both tests were set to 0.05.

To correlate species abundances to the clinical features for HIV-infected individuals, we performed a generalized linear model (GLM) by setting the clinical variables as the response variable and the species matrix as the predictors using the cv. glmnet function in the “glmnet” R package. The results were validated using the Spearman correlation index.

The linear discriminant analysis (LDA) effect size (LEfSe) algorithm was applied to identify biomarkers of the microbiota composition from the different groups [27]. Default parameters were used for significance (*p*-value <0.05) and linear discriminant analysis threshold (>2.0).

To control for the false discovery rate, we corrected the statistical tests adjusting all *p*-values using the Benjamini–Hochberg correction (library “stats”, function “p.adjust”) (*q*-value).

To determine if the sample size of the groups before and after prebiotics offered sufficient statistical power to detect differences through a bilateral Student’s *t*-test with a significance of 5%, we applied the function “pwr.t2n.test” (two-sample *t*-tests with unequal sample sizes) from the pwr R package. We verified that the estimated average sample size needed to achieve 90% and 85% power was similar to the real sample size that we had for the groups before and after prebiotic intervention, respectively (Tables S2–S5, Supplementary Materials).

### 2.6. Co-Occurrence of Bacterial Taxa in Salivary and Gut Microbiota

We performed a cluster analysis at 100% similarity to search for identical 16S rRNA sequences in paired samples of saliva (S)/feces (F). For each OTU, we counted how many read-pairs (S–F) from the same individual were contained in that OTU. In order to quantify to what extent the co-occurrence events were biologically driven or, on the contrary, simply random, we estimated for each OTU the following log-likelihood ratio, log2(Prob(S,F)/(Prob(F) × Prob(S)), which was based on the Bayes formula. Thus, the OTUs presenting such a log-likelihood ratio above zero would represent a biological phenomenon. However, to be conservative, we considered the OTUs with a log-likelihood ratio (log2(Prob(S,F)/Prob(F) × Prob(S))) higher than 0.5 as biological events.

## 3. Results

### 3.1. General Features of the Patients and Samples

A total of 32 participants completed the six-week-long prebiotic intervention: 7 controls, 6 INR, 10 IR, and 9 VU (Figure S1, Supplementary Materials). Thus, we analyzed 85 salivary samples corresponding to 53 salivary samples at baseline (F1 samples) and 32 salivary samples collected after the prebiotic intervention (F2 samples). We also analyzed the stools from 52 subjects at baseline and from 35 individuals after prebiotics (Table 1).

We determined at baseline the differences between the four groups (IR, INR, VU, and HIV−) in plasma metabolic profile, T-cell markers, thymic function, endothelial function, bacterial translocation, inflammation, and thrombosis (Table 2). We found significant differences between healthy controls and HIV-infected groups (INR, IR, VU) in T-cell markers (T helper CD4+ cells/μL and CD8+ cells/μL) and thymic function measured as signal joint/beta T-cell receptor excision circles (sj/β-TREC) ratio (Table 2).

We also determined the clinical variables after prebiotics (Table 2). We observed a decrease in both bacterial translocation markers and in T-cell activation markers for the four groups.

### 3.2. Salivary Microbiota Analysis in HIV-Infected Individuals

To characterize the salivary microbiota composition at baseline, we sequenced 53 samples obtaining 1,878,045 high-quality 16S rRNA sequences with an average of 42,682 sequences per sample.

**Figure 1:**
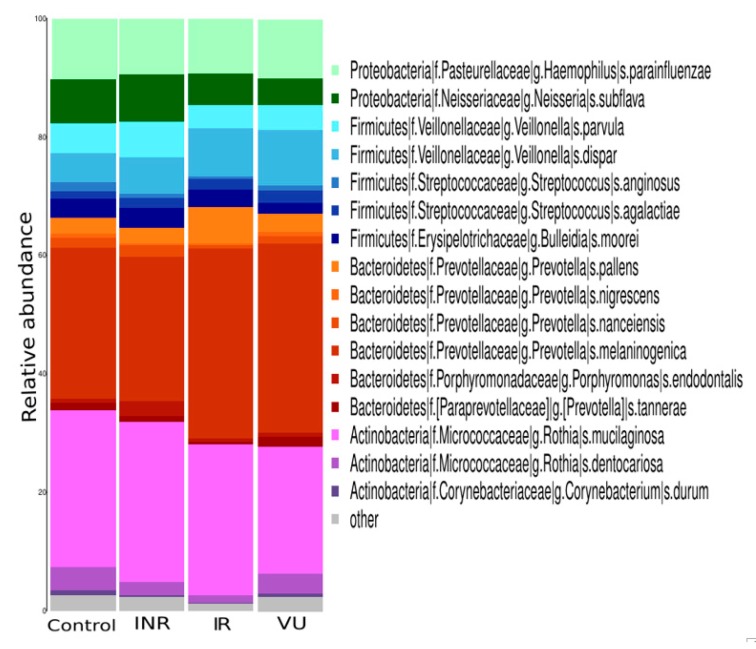
Oral microbiota composition in HIV-infected groups and controls. Species distribution of the salivary microbiota in each group. INR, immunological antiretroviral therapy (ART) non-responders; IR, immunological ART responders; VU, viremic untreated.

The taxonomic analysis (Figure 1) revealed that the most abundant bacteria were similar in all the groups: *Corynebacterium* (*C. durum*) and *Rothia* (*R. mucilaginosa* and *R. dentocariosa*) from the Actinobacteria phylum, *Porphyromonas* (*P. endodontalis*) and *Prevotella* (*P. pallens*, *P. nigrescens*, *P. nanceiensis*, *P. melaninogenica*, and *P. tannerae*) from the Bacteroidetes, *Bulleidia* (*B. moorei*), *Streptococcus* (*S. anginosus* and *S. agalactiae*), and *Veillonella* (*V. parvula* and *V. dispar*) from the Firmicutes, and *Neisseria* (*N. subflava*) and *Haemophilus* (*H. parainfluenzae*) from the Proteobacteria.

**Figure 2:**
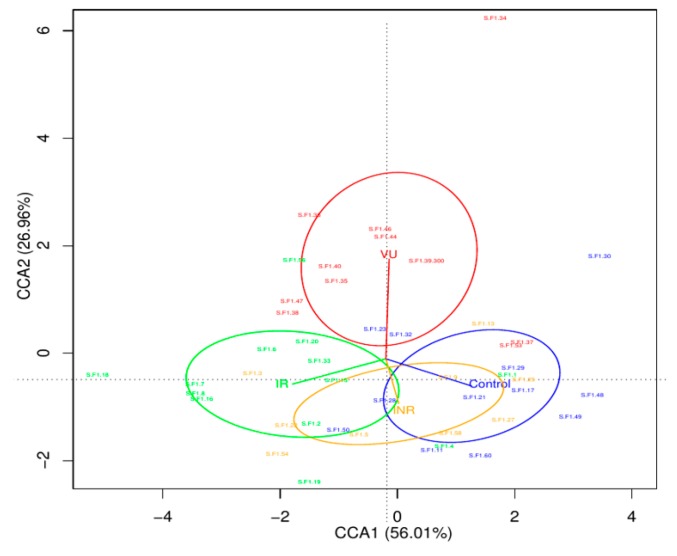
Canonical correspondence analysis (CCA) based on operational taxonomic unit (OTU) abundance. INR, immunological ART non-responders (yellow); IR, immunological ART responders (green); VU, viremic untreated (red); and controls (blue).

We applied the ADONIS test to evaluate whether the HIV status is a factor that influences the microbiota structure. This test validated (*p*-value = 0.04) the compositional differences observed at the OTU level in the CCA analysis (Figure 2). This ordination technique showed that the first axis, explaining 56.01% of variability, separated the IR and control groups from the rest, while the second axis, explaining 26.96% of variability, split the VU group from the other three groups. To identify the OTUs that explained the differences between the four groups, we used LEfSe analysis. We found that the VU group presented as biomarkers *Streptococcus agalactiae* and *V. parvula*, while IR had different species of *Streptococcus.* OTUs belonging to Actinobacteria were characteristic for INR and control groups (Figure S2a, Supplementary Materials). We also applied LefSe analysis between HIV+ and HIV− groups (Figure S2b, Supplementary Materials). We found that the HIV-associated salivary microbiota presented a significantly higher abundance of potential pathogens as *S. agalactiae* (*p*-value = 0.0091), *Corynebacterium durum* (*p*-value = 0.013), and OTUs assigned to species of *Prevotella* (*p*-value = 0.046), *Leptotrichia* (*p*-value = 0.048), *Tannerella* (*p*-value = 0.004), and *Catonella* (*p*-value = 0.028).

**Figure 3:**
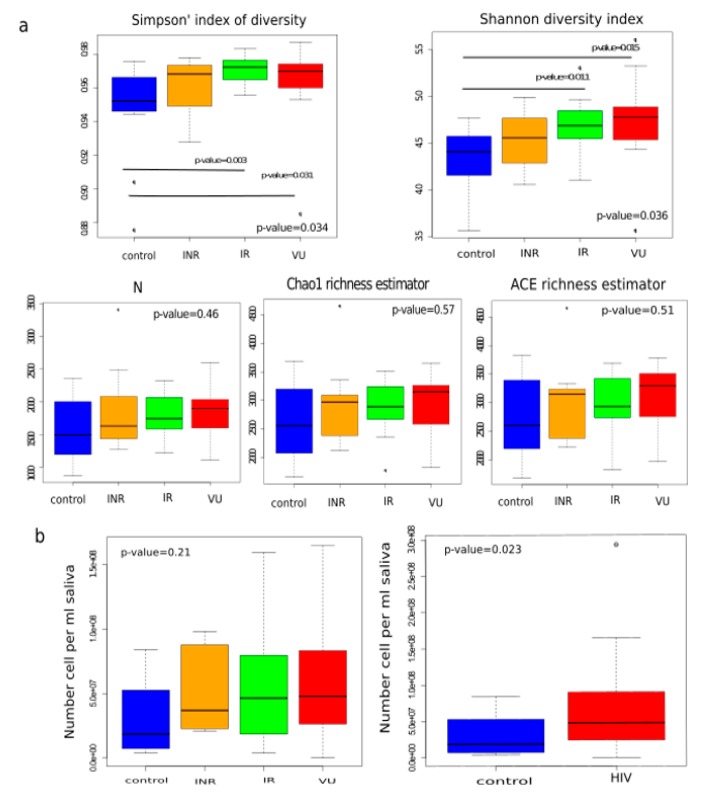
Alpha diversity and bacterial load in saliva. (**a**) Simpson’s index of diversity, Shannon diversity index, number of OTUs observed (*N*), Chao1 richness estimator, and Abundance-based Coverage Estimator (ACE) in the four groups. (**b**) Bacterial load expressed as number of cells per mL of saliva in the four groups. and bacterial load expressed as number of cells per mL of saliva in HIV-infected individuals (HIV) and controls. To calculate differences across groups, we used the Kruskal–Wallis test (*p*-value = 0.05) and, for differences between groups, we used the Wilcoxon rank-sum test (*p*-value = 0.05). INR, immunological ART non-responders (yellow); IR, immunological ART responders (green); VU, viremic untreated (red); and controls (blue).

When we analyzed the diversity (Simpson’s index of diversity and Shannon index) and richness (Chao1 and ACE estimator) of the salivary microbiota in each group at the OTU level, we observed that the VU group presented the highest value for these two parameters, followed by the IR group (Figure 3a). Moreover, both groups were significantly more diverse than the control group. Additionally, the bacterial load in salivary samples from HIV-infected patients was higher than in HIV− samples (Figure 3b). The impaired immunological system of VU individuals could explain the higher number of bacterial cells per mL of saliva and the higher diversity parameters.

### 3.3. Effect of Prebiotics on Salivary Microbiota

**Figure 4:**
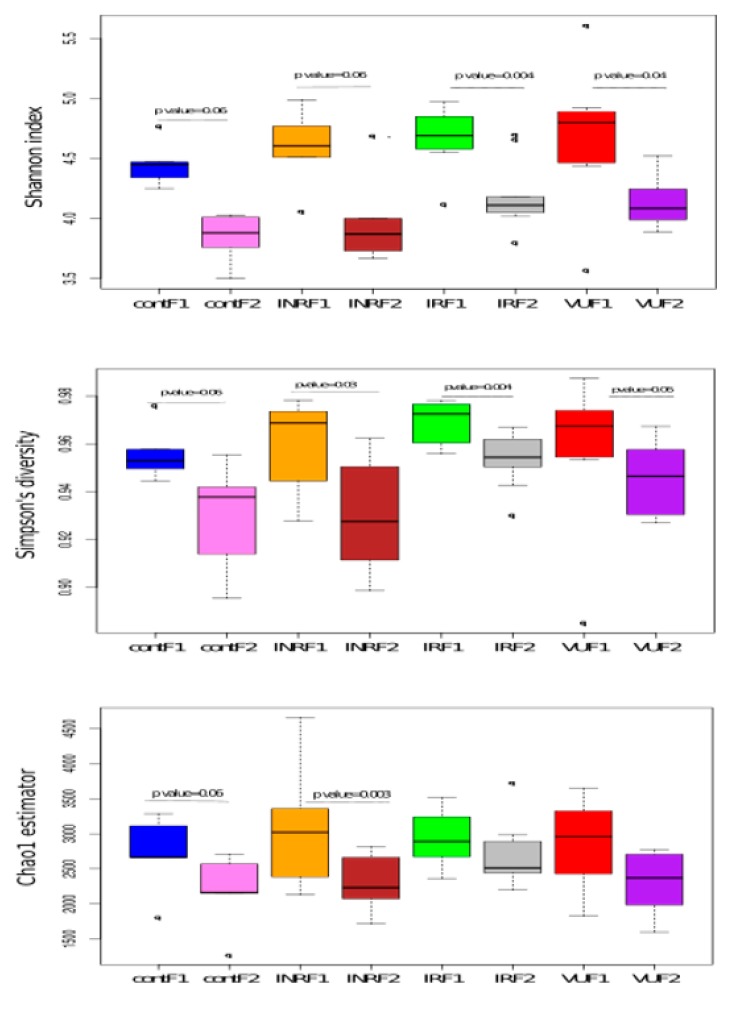
Alpha diversity before and after prebiotic intervention. Shannon diversity index, Simpson’s index of diversity, and Chao1 richness estimator in the four groups. To calculate differences between groups, we used the Wilcoxon rank-sum test (*p*-value = 0.05). F1, at baseline; F2, after prebiotics. INR, immunological ART non-responders; IR, immunological ART responders; VU, viremic untreated; cont, controls.

After nutritional intervention, the IR and VU groups presented higher diversity parameters (Simpson’s index of diversity, Shannon index, and Chao1 richness estimator) than INR and control individuals (Figure 4), as it occurred at baseline. To assess the effect of prebiotics on alpha diversity, we compared F1 and F2 samples in each group. Interestingly, we observed a drastic decrease in both diversity and richness parameters after prebiotic intervention with a similar fold change for the four groups (Figure 4).

**Figure 5:**
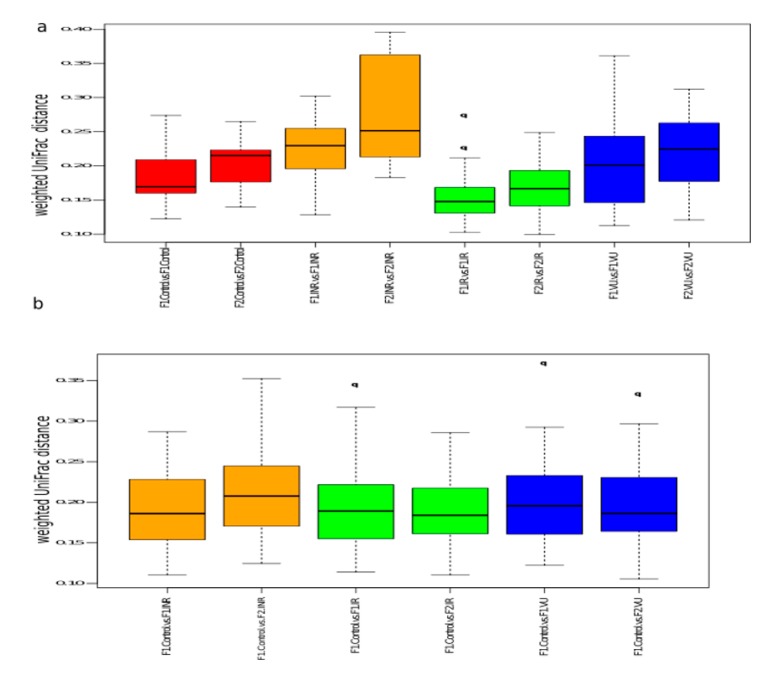
(**a**) Beta diversity at baseline (F1) and after prebiotics (F2) for control (red), INR (orange), IR (green), and VU (blue) groups. (**b**) Weighted UniFrac distances between Control.F1 and F1 or F2 of INR (orange), IR (green), and VU (blue). INR, immunological ART non-responders; IR, immunological ART responders; VU, viremic untreated.

To evaluate the changes in the overall community structure in each group, we used the within-group weighted Unifrac distances. We found that prebiotic intervention modified the microbiota structure in all the groups, increasing the heterogeneity (Figure 5a). Then, we hypothesized that prebiotics could ameliorate the HIV-associated dysbiosis observed in salivary microbiota. To address this point, we considered the HIV− group at baseline as a healthy reference (F1.Control) and we calculated the weighted UniFrac distances between F1.Control and F1 or F2 for each group. We found that the weighted UniFrac distance between the F1.Control and F1.VU groups was slightly higher than that between F1.Control and F2.VU, which suggested a trend of the microbiota composition toward the microbiota of the healthy reference (Figure 5b and Figure S3, Supplementary Materials). In the IR group, we did not observe a clear approximation to the healthy reference after prebiotics. However, we observed an increase of the distance to the F1.Control for the F2.INR group (Figure 5b and Figure S3, Supplementary Materials).

**Figure 6:**
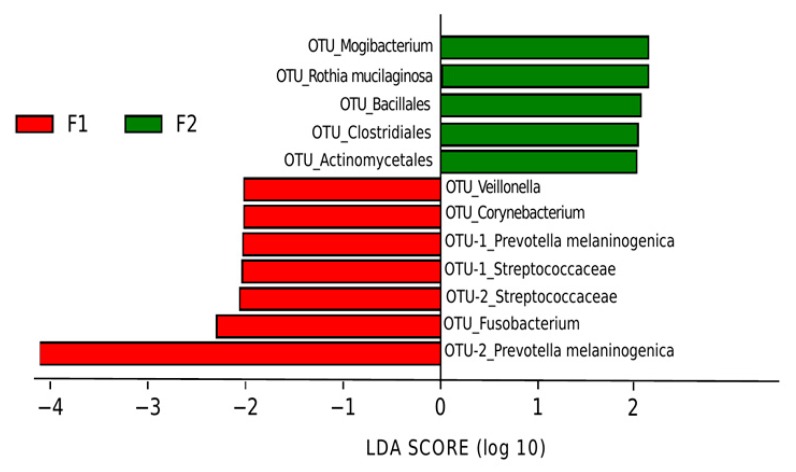
Linear discriminative analysis (LDA) effect size (LEfSe) at the OTU level in viremic untreated group (VU) before (F1) and after (F2) prebiotic intervention. LDA scores for the significant taxa in F2 are represented on the positive scale (green), and LDA-negative scores represent enriched taxa in F1 (red).

Then, we used LEfSe analysis to identify the OTUs that presented significant differences between F1 and F2 in the four groups. Interestingly, we only found a change of microbiota composition in the VU group (Figure 6). Thus, the viremic ART-naïve individuals, after prebiotics, showed a microbiota enriched in the Actinobacteria *Rothia mucilaginosa,* a commensal bacterium of the oral cavity associated with dental and periodontal health. In addition, although the genus *Mogibacterium* was increased in F2, we found after prebiotics a depletion of other potential pathogens such as *Corynebacterium*, *Fusobacterium*, or *Prevotella melaninogenica*.

### 3.4. Association between Salivary Microbiota and Clinical Markers before and after Prebiotic Intervention

GLM analysis showed significant correlations between specific bacterial members of salivary microbiota and markers of bacterial translocation, adaptive immunity, and plasma metabolites (Table 3). At baseline, *Streptococcus anginosus* correlated with CD4+ T cells, *Veillonella parvula* with CD4+ CD25+ T cells, and *Prevotella pallens, P. copri*, and *P. nigrencens* with markers of adaptive immunity such as CD4+ CD25+ T cells, CD4+ CD57+ T cells, and CD4+ HLADR+ (Human Leukocyte Antigen –DR isotype) CD38+ T cells, respectively. Also, another major species *Haemophilus parainfluenzae* presented a significant positive correlation with the concentration of creatinine in plasma. Additionally, we found significant correlations between minor species and other clinical variables. After prebiotic intervention, we observed that different species of *Lactobacillus* and *Streptococcus* correlated negatively with inflammatory marker interleukin-6 (IL-6) and bacterial translocation (soluble CD14, sCD14). Also, *Ruminococcus gnavus* correlated negatively with T-cell activation markers.

### 3.5. Comparison between Salivary and Gut Microbiota: Bacterial Co-Occurrence

Firstly, we compared salivary and fecal bacterial populations by applying a PCoA analysis, in which, as expected, the two microbiotas presented highly different structures (Figure S4, Supplementary Materials). On the basis of the connection between the oral cavity and the gut, we explored the bacterial co-occurrence in both environments. To this aim, we searched for 100% identical 16S rRNA sequences in salivary and feces samples in each group (control, INR, IR, VU) at baseline and after prebiotic intervention, which yielded 330,120 and 272,622 total OTUs, respectively. We selected those OTUs that presented at least one pair of saliva and feces sequences from the same individual, representing the co-occurrence events. As we observe in Table S1 (Supplementary Materials), the co-occurrence events took place in the four groups, but they were not very frequent. This result is congruent with the different microbiota structure that is present in each habitat. 

**Figure 7:**
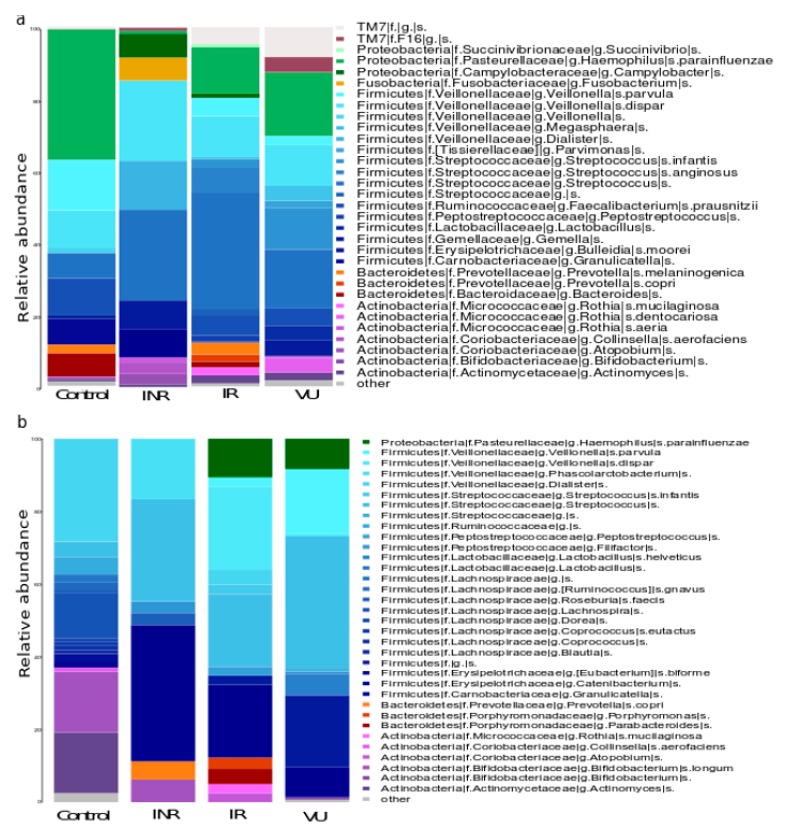
Taxa distribution of co-occurrences in each group: (**a**) at baseline; (**b**) after prebiotics. INR, immunological ART non-responders; IR, immunological ART responders; VU, viremic untreated.

We observed a decrease in the frequency of co-occurrences after prebiotics in the HIV-infected patients (Table S1, Supplementary Materials). At baseline, the bacterial composition analysis showed that the more frequent co-occurrences were detected for the major bacterial genera and species of the salivary microbiota such as *Haemophilus parainfluenzae*, *Streptococcus*, and *Veillonella* (*V. dispar*, *V. parvula*) (Figure 7). Surprising, we also found co-occurrences of colonic bacteria such as *Faecalibacterium prausnitzii*, *Dialister*, and *Bifidobacterium.* The clustering analysis (Figure S5, Supplementary Materials) showed that IR and VU grouped together before and after prebiotic intervention, while the control presented a differential profile. Moreover, we identified specific bacterial taxa that only occurred in each of the groups. After prebiotics, we observed an increase of co-occurrences of *Lactobacillus*, *Streptococcus*, and *Ruminococcus* genera.

## 4. Discussion

In the last few years, high-throughput sequencing allowed deep and holistic studies of complex environments such as the human body. The oral cavity is no exception, with more than 700 species detected in the mouth microbiome [1,28]. Furthermore, other studies revealed the oral microbiota as a clear determinant of systemic inflammation and cardiovascular risk [28,29,30].

Currently, HIV infection is considered as a chronic inflammatory disease that alters the interplay between the gut microbiota and immune system. HIV-associated gut dysbiosis was studied in depth, and different nutritional approaches with prebiotics, probiotics, and synbiotics showed changes in immunity parameters and microbiota composition. However, few works addressed the dysbiosis of the oral microbiota in HIV infection and, to our knowledge, no studies on intervention with prebiotics were performed.

According to previous works [18,19,20,21,22], differences in the relative abundance of several bacterial taxa between HIV-infected subjects and healthy controls were observed, but the overall salivary microbiota structure was similar in all groups. However, the ordination analysis showed that the HIV infection had a considerable impact on the salivary microbiota. Unlike Li et al. [18], we detected an increase of diversity parameters for salivary microbiota in HIV-infected patients, mainly in viremic ART-naïve subjects (VU group) as we previously described in HIV-associated fecal microbiota [14]. A similar effect of HIV infection on bacterial community diversity was also described in the HIV-associated genital microbiota of women with vaginosis [31]. Furthermore, Presti et al. [22] described a correlation between viral suppression and decrease of diversity in HIV-associated salivary microbiota. Thus, the impairment of the immune system produced by HIV infection allowed the growth and colonization of opportunistic bacteria and pathogens in the oral cavity, suggesting that the composition of the oral bacterial community could reflect the HIV status. Although, we observed correlations between the microbiota and biomarkers of immunity, bacterial translocation, and thymic function, we could not establish a causal influence between the oral microbiome and the immune system.

In the last few years, the modulation of the microbiome by probiotics and prebiotics appeared as a promising tool to drive a dysbiotic microbiota toward a healthy status. In oral health, probiotics interventions were applied in the prevention of different diseases such as dental caries, gingivitis, and periodontitis [1,2,10]. However, few studies focused on the application of prebiotics. A recent work screened potential nutritional compounds for the stimulation of commensal bacteria growth with beneficial effects on oral health [8,9]. Even though different surveys applied prebiotics, probiotics, or synbiotics to modify the dysbiotic gut microbiome in HIV patients [12,13,14,15,16,17], no such studies were performed for oral HIV-associated microbiota. In the present work, we evaluated the modulatory effect of six weeks of prebiotic intervention on the salivary microbiota in the cohort previously described by Serrano-Villar et al. [14]. We are aware that the low number of enrolled patients hinders the interpretation of the effects of prebiotics on the salivary microbiota and demands caution in conclusions. In spite of this, our survey is the first study on the prebiotic modulation of the salivary microbiota in HIV-infected patients with diverse immunopathogenesis.

The decrease in alpha diversity of the saliva bacterial community after dietary intervention suggested a growth stimulation of specific bacteria that could inhibit pathogenic species. Although the prebiotics changed the beta diversity of salivary bacterial communities, no clear restoration of a healthy microbiota was detected. Our findings suggested that the response to prebiotics might depend on the HIV status. In fact, in the viremic ART-naïve group, we observed an approximation to the healthy condition, but contradictory responses were observed in ART-treated individuals. Furthermore, in accordance with what we previously described in the gut microbiota [14], the modulation of prebiotics was more apparent in the viremic ART naïve group (VU) with an enrichment in commensal bacteria and a depletion in potential pathogens. To validate our results and to extend the understanding of the mechanisms of action of prebiotics on the oral microbiota and their relationship with the gut microbiome, more studies will be needed with larger number of patients.

Due to the anatomical and physiological link between saliva and the gastrointestinal tract, we tackled the potential bacterial exchange between the salivary and gut microbiome. Several studies identified oral bacteria in the fecal microbiota in different diseases such as Crohn’s disease, liver cirrhosis, or colorectal carcinoma [32,33,34,35,36], suggesting a role of the oral microbiota in the inflammatory process. Recently, Atarashi et al. [37] showed that the oral genus, *Klebsiella*, colonized the gut inducing intestinal inflammation. However, the knowledge of the underlying basis for the bacterial exchange between both habitats is scarce [38,39]. In this work, we assessed the bacterial co-occurrences between salivary and fecal HIV-associated microbiota. The major salivary taxa would flow through the intestinal tract and would survive in the colon, albeit at a low abundance. Furthermore, in accordance with Franzosa et al. [39], we also detected co-occurrences of gut commensal bacteria such as *Dialister* and *Faecalibacterium* in healthy and HIV-infected individuals, suggesting a potential bidirectional flux for some bacteria. Interestingly, the bacterial exchange profile of HIV-infected subjects clustered separately from those of healthy individuals, which may suggest that the HIV infection modifies the bacterial exchange between the oral and gut microbiome. A better understanding of the interplay between the oral and gut microbiome may encourage the design of new nutritional strategies to modulate dysbiotic microbiota in inflammatory diseases.

## Figures and Tables

**Table 1:** Saliva and fecal samples used in the study.

Saliva F1 ^a^	Saliva F2 ^b^	Feces F1 ^a^	Feces F2 ^b^	HIV Group ^c^
S-F1-11		F1-11		Control
S-F1-17		F1-17		Control
S-F1-21		F1-21		Control
S-F1-23	S-F2-23	F1-23	F2-23	Control
S-F1-28	S-F2-28	F1-28	F2-28	Control
S-F1-29	S-F2-29	F1-29	F2-29	Control
S-F1-30		F1-30		Control
S-F1-31		F1-31		Control
S-F1-32		F1-32		Control
S-F1-45	S-F2-45	F1-45	F2-45	Control
S-F1-48		F1-48		Control
S-F1-49	S-F2-49	F1-49	F2-49	Control
S-F1-50	S-F2-50	F1-50	F2-50	Control
S-F1-60	S-F2-60	F1-60	F2-60	Control
S-F1-13	S-F2-13	F1-13	F2-13	INR
S-F1-22	S-F2-22	F1-22	F2-22	INR
S-F1-25	S-F2-25	F1-25	F2-25	INR
S-F1-27	S-F2-27	F1-27	F2-27	INR
S-F1-3		F1-3		INR
S-F1-5	S-F2-5	F1-5	F2-5	INR
S-F1-54		F1-54		INR
S-F1-58		F1-58	F2-58	INR
S-F1-9	S-F2-9	F1-9	F2-9	INR
S-F1-1	S-F2-1	F1-1		IR
S-F1-10		F1-10		IR
S-F1-12		F1-12		IR
S-F1-14	S-F2-14	F1-14	F2-14	IR
S-F1-15		F1-15	F2-15	IR
S-F1-16	S-F2-16	F1-16	F2-16	IR
S-F1-18	S-F2-18	F1-18	F2-18	IR
S-F1-19		F1-19	F2-19	IR
S-F1-2	S-F2-2	F1-2	F2-2	IR
S-F1-20	S-F2-20	F1-20	F2-20	IR
S-F1-24		F1-24		IR
S-F1-26		F1-26		IR
S-F1-33	S-F2-33	F1-33	F2-33	IR
S-F1-4	S-F2-4	F1-4	F2-4	IR
S-F1-56		F1-56		IR
S-F1-6		F1-6		IR
S-F1-7	S-F2-7	F1-7	F2-7	IR
S-F1-8	S-F2-8	F1-8	F2-8	IR
S-F1-34				VU
S-F1-35	S-F2-35	F1-35	F2-35	VU
S-F1-36	S-F2-36	F1-36	F2-36	VU
S-F1-37		F1-37		VU
S-F1-38		F1-38	F2-38	VU
S-F1-39	S-F2-39	F1-39	F2-39	VU
S-F1-40	S-F2-40	F1-40	F2-40	VU
S-F1-44	S-F2-44	F1-44	F2-44	VU
S-F1-46	S-F2-46	F1-46	F2-46	VU
S-F1-47	S-F2-47	F1-47	F2-47	VU
S-F1-51	S-F2-51	F1-51	F2-51	VU
S-F1-53	S-F2-53	F1-53	F2-53	VU

^a^: baseline level, ^b^: after prebiotics, ^c^: INR, immunological antiretroviral therapy (ART) non-responder; IR, immunological ART responder; VU, viremic untreated.

**Table 2:** Clinical variables at baseline and after prebiotic intervention in studied subjects.

	Control		INR		IR		VU					
	Baseline	Prebiotics	Baseline	Prebiotics	Baseline	Prebiotics	Baseline	Prebiotics	*p*.value Baseline ^a^	Adj.fdr *p*.value Baseline ^a^	*p*.value Prebiotics ^b^	Adj.fdr *p*.value Prebiotics ^b^
**Metabolic profile in plama**												
Glucose (mg/dL)	91 (88–94)	90(83–98)	93(89–96)	94(88–108)	93(89–97)	90(86–99)	85(82–87)	92(85–94)	0.0615	0.1285	0.6181	0.6476
Creatinine (mg/dL)	0.97 (0.82–1)	0.91 (0.84–0.99)	1(0.95–1)	1.1 (0.96–1.2)	1(0.96–1.1)	0.97 (0.93–1.1)	1(0.94–1.2)	0.96(0.89–1)	0.3194	0.4081	0.1024	0.1733
Total cholesterol (mg/dL)	207 (160–220)	149 (136–192)	148 (145–198)	155 (135–185)	185 (153–208)	190 (156–220)	158 (147–184)	164 (160–177)	0.0924	0.1771	0.2299	0.3161
HDL-cholesterol (mg/dL)	54(49–75)	55(52–76)	50(44–57)	49(43–61)	53(47–58)	56(48–60)	48(41–53)	49(40–53)	0.3141	0.4081	0.3028	0.3918
LDL-cholesterol (mg/dL)	116 (82–134)	89 (74–126)	78 (71–116)	81 (69–99)	105 (93–119)	115 (89–132)	88 (86–100)	94 (86–103)	0.1423	0.2517	0.3867	0.4477
Triglycerides (mg/dL)	80 (66–161)	67(54–76)	102(87–147)	97(75–120)	102(81–127)	104(75–152)	107(63–158)	110 (103–135)	0.9266	0.9687	0.9188	0.9188
**T cell markers**												
CD4+T-cell counts (cells/μl)	716 (601–862)	742 (629–880)	271 (204–321)	289 (227–319)	561 (426–667)	534 (432–668)	521 (385–738)	549 (458–645)	0.0001	0.0007	0.0031	0.0115
%HLADR+ CD38+	1(0.7–1.1)	1.2 (0.89–1.4)	3.4(3–4)	3.3(2.6–3.8)	2.3(1.8–2.6)	1.9(1.5–2)	3.8(3.6–7.8)	3.4(2.8–3.6)	0.0000	0.0000	0.0000	0.0001
%CD25+	2.6(2–4)	2.6(2.1–3.2)	6.1(5.5–8.3)	6.6(6–7.5)	4.7(4.2–6.6)	4.5(4.2–6.4)	8.2(5.4–10)	6.2(4.6–7.3)	0.0028	0.0108	0.0013	0.0058
%CD57+	3.2 (2.2–5.9)	5.1(2.4–5.9)	11(3.7–12)	8.3(4.3–11)	5.7(4–8.6)	6.8(3.3–10)	13(5.7–22)	9.3(8.7–22)	0.0211	0.0485	0.0498	0.1095
CD8+ T-cell counts (cells/μl)	403 (306–552)	429 (308–541)	404 (377–695)	434 (226–566)	606 (480–730)	532 (436–670)	1004 (794–1114)	1075 (829–1271)	0.0079	0.0228	0.0140	0.0439
%HLADR +CD38+	2(1.2–2.1)	1.5(0.6–1.6)	4.8(4.4–6.3)	4.9(3.7–8)	3.3(2.6–8.3)	4.8(3.8–5.7)	13(10–17)	10(8.1–11)	0.0000	0.0000	0.0001	0.0004
%CD25+	0.33 (0.095–0.41)	0.19 (0.13–0.23)	0.73 (0.57–1.6)	0.84 (0.47–1.2)	0.47 (0.32–0.74)	0.46 (0.26–0.6)	0.95 (0.93–1.1)	0.77 (0.72–0.85)	0.0022	0.0102	0.0184	0.0507
%CD57+	24(16–46)	18(18–57)	27(18–34)	29(19–34)	26(18–42)	31(21–47)	42(39–55)	46(36–56)	0.2309	0.3540	0.3801	0.4477
CD4/CD8 ratio	1.5 (1.2–1.7)	1.7 (1.3–2)	0.65 (0.36–0.89)	0.88 (0.36–2.2)	1(0.91–1.2)	1.2(1.1–1.3)	0.54 (0.45–0.74)	0.56 (0.51–0.62)	0.0001	0.0006	0.0011	0.0058
**Thymic function**												
sj/β-TREC ^c^ ratio	9(8.2–9.5)	8.8(7.1–8.9)	7.8(7.6–8.4)	8.2 (7.9–8.8)	8.2(7.7–9.2)	9.1(8.3–9.6)	8.7(8.3–9)	8.4 (7.4–8.8)	0.0195	0.0485	0.5480	0.6029
**Endotelial function**												
ADMA ^d^ (μM/L)	1.1 (1.1–1.1)	1(0.9–1.1)	0.97 (0.86–1.2)	1.2(1–1.3)	0.97 (0.96–1.1)	0.99(0.95–1)	0.96 (0.92–1.1)	1.1(1–1.2)	0.2506	0.3602	0.2074	0.3042
**Bacterial translocation**												
BPI ^e^ (ng/mL)	35(12–35)	8.3(2.6–9.1)	13(4.1–49)	3(2.3–5.7)	28(1.3–114)	6(1.8–8.6)	17(5.3–60)	9.8(5.3–17)	0.9768	0.9768	0.0000	0.0002
SCD14 ^f^ (ng/mL)	1424 (1254–1484)	1272 (1162–1362)	1636 (1454–1999)	1894 (1641–2402)	1663 (1483–1732)	1610 (1251–1750)	1552 (1360–1613)	1507 (1295–1721)	0.1587	0.2607	0.0463	0.1095
**Inflammation**												
IL6 ^g^ (pg/mL)	2(2–4.1)	2(2–2)	2(2–2.2)	7.3(5.3–8.1)	2(2–2)	2(2–2.7)	2(2–2)	2(2–4.3)	0.3811	0.4614	0.0665	0.1329
Hs-CRP ^h^ (mg/L)	0.095 (0.055–0.3)	0.07 (0.035–0.22)	0.11 (0.06–0.15)	0.57 (0.28–1.3)	0.18 (0.09–0.29)	0.19 (0.062–0.34)	0.09 (0.053–0.18)	0.15 (0.07–0.26)	0.6838	0.7489	0.0835	0.1530
**Thrombosis**												
Dimers-D (ng/mL)	180 (120–274)	240 (209–304)	202 (113–306)	261 (237–290)	215 (176–293)	202 (184–232)	280 (202–322)	238 (216–397)	0.4032	0.4637	0.1428	0.2244

^a^, Analysis was performed using Kruskal–Wallis test to compare median values across all groups at baseline. ^b^, Analysis was performed using Kruskal–Wallis test to compare median values among control at baseline and HIV-infected patients after prebiotics. The *p*-value represents probability at α = 0.05. The *p*-values were adjusted by the Benjamini–Hochberg method. ^c^, signal joint/beta T-cell receptor excision circles ratio; ^d^, asymetric dymethilarginine; ^e^, bactericidal permeability increasing protein; ^f^, soluble CD14; ^g^, interleukin-6; ^h^, high-sensitivity C-reactive protein. INR, immunological ART non-responders; IR, immunological ART responders; VU, viremic untreated.

**Table 3:** Correlations between saliva microbiota and clinical parameters at baseline and after treatment. Analysis was performed using Kruskal–Wallis test to compare median values across all groups. The *p*-value is the probability at α = 0.05. The *p*-values were adjusted by the Benjamini–Hochberg method.

**Correlations Before Prebiotic Intervention**				
**Clinical Variable**	**Taxa**	**Spearman Correlation Index**	***p*.value**	**Adj.fdr_*p*.value**
**Plasma Metabolite**				
Creatinine	*Faecalibacterium prausnitzii*	0.5893	0.0004	0.0344
Creatinine	*Haemophilus parainfluenzae*	0.5670	0.0007	0.0376
Creatinine	*Actinobacillus porcinus*	0.4944	0.0040	0.0710
HDL-cholesterol	*Prevotella tannerae*	−0.4664	0.0071	0.1025
HDL-cholesterol	*Lactobacillus coleohominis*	−0.4136	0.0186	0.1346
HDL-cholesterol	*Pyramidobacter piscolens*	−0.3985	0.0239	0.1356
HDL-cholesterol	*Lactobacillus reuteri*	−0.3955	0.0250	0.1356
HDL-cholesterol	*Pasteurella multocida*	−0.3851	0.0295	0.1356
**T cell markers**				
CD4+T cell	*Streptococcus* *anginosus*	−0.3965	0.0247	0.1356
CD4+T cell	*Pyramidobacter piscolens*	−0.3682	0.0381	0.1356
CD4+ CD25+T cell	*Veillonella parvula*	0.4170	0.0176	0.1346
CD4+ CD25+T cell	*Prevotella pallens*	−0.3736	0.0352	0.1356
CD4+ CD57+T cell	*Prevotella copri*	0.3864	0.0289	0.1356
CD4+ CD57+T cell	*Pasteurella multocida*	0.3587	0.0438	0.1356
CD4+ HLADR+ CD38+T cell	*Prevotella nigrescens*	0.3592	0.0442	0.1356
**Thymic function**				
sj/β-TREC ratio ^a^	*Actinomyces hyovaginalis*	0.5852	0.0004	0.0344
sj/β-TREC ratio	*Streptococcus sobrinus*	−0.5077	0.0030	0.0680
sj/β-TREC ratio	*Streptococcus agalactiae*	0.4780	0.0062	0.0975
sj/β-TREC ratio	*Pyramidobacter piscolens*	−0.3710	0.0366	0.1356
Bacterial translocation				
BPI ^b^	*Streptobacillus moniliformis*	0.3550	0.0462	0.1356
**Correlations After Prebiotic Intervention**				
**Clinical Variable**	**Taxa**	**Spearman Correlation Index**	***p*.value**	**Adj.fdr *p*.value**
**Plasma Metabolite**				
Creatinine	*Campylobacter rectus*	0.3741	0.0349	0.0689
Glucose	*Actinomyces* *hyovaginalis*	−0.4022	0.0225	0.0689
Glucose	*Collinsella aerofaciens*	0.3575	0.0446	0.0689
HDL-cholesterol	*Lactobacillus coleohominis*	−0.3839	0.0301	0.0689
HDL-cholesterol	*Prevotella melaninogenica*	0.3761	0.0339	0.0689
LDL-cholesterol	*Pasteurella multocida*	−0.3893	0.0276	0.0689
Triglycerides	*Pasteurella multocida*	−0.4501	0.0098	0.0689
Triglycerides	*Lactobacillus coleohominis*	0.4211	0.0164	0.0689
Triglycerides	*Lactobacillus reuteri*	0.3714	0.0364	0.0689
**T cell markers**				
CD4+T cell	*Ruminococcus gnavus*	0.5321	0.0017	0.0283
CD4+ HLADR+ CD38+T cell	*Bifidobacterium adolescentis*	0.3928	0.0262	0.0689
CD4+ HLADR+ CD38+T cell	*Ruminococcus gnavus*	−0.3618	0.0419	0.0689
CD4+ HLADR+ CD38+T cell	*Capnocytophaga ochracea*	0.3590	0.0436	0.0689
CD4+ CD25+T cell	*Ruminococcus gnavus*	−0.5393	0.0014	0.0283
CD4+ CD25+T cell	*Actinobacillus porcinus*	0.3916	0.0267	0.0689
CD4+ CD25+T cell	*Prevotella_intermedia*	0.3901	0.0273	0.0689
CD4+ CD25+T cell	*Selenomonas_noxia*	−0.3821	0.0309	0.0689
CD4+ CD57+T cell	*Neisseria_subflava*	−0.4621	0.0078	0.0689
CD4+ CD57+T cell	*Atopobium_rimae*	0.4522	0.0094	0.0689
CD4+ CD57+T cell	*Haemophilus_parainfluenzae*	−0.4359	0.0133	0.0689
CD4+ CD57+T cell	*Veillonella dispar*	0.4245	0.0162	0.0689
CD4+ CD57+T cell	*Pasteurella multocida*	−0.4036	0.0220	0.0689
CD4+ CD57+T cell	*Prevotella intermedia*	0.3979	0.0241	0.0689
CD4+ CD57+T cell	*Bifidobacterium adolescentis*	0.3612	0.0423	0.0689
CD8+T cell	*Capnocytophaga ochracea*	0.3983	0.0240	0.0689
CD8+ CD25+T cell	*Pseudomonas veronii*	−0.4773	0.0057	0.0672
CD8+ CD25+T cell	*Bulleidia moorei*	−0.3796	0.0321	0.0689
CD8+ CD25+T cell	*Bifidobacterium longum*	−0.3699	0.0372	0.0689
CD8+ CD57+T cell	*Pasteurella multocida*	−0.5469	0.0012	0.0283
CD8+ CD57+T cell	*Pasteurella multocida*	−0.5469	0.0012	0.0283
CD8+ CD57+T cell	*Flexispira rappini*	−0.3800	0.0319	0.0689
CD8+ CD57+T cell	*Flexispira rappini*	−0.3800	0.0319	0.0689
CD8+ CD57+T cell	*Neisseria subflava*	−0.3598	0.0431	0.0689
CD8+ CD57+T cell	*Streptobacillus moniliformis*	−0.3577	0.0444	0.0689
CD4/CD8 ratio	*Ruminococcus gnavus*	0.4574	0.0085	0.0689
**Endotelial function**				
ADMA ^c^	*Faecalibacterium prausnitzii*	0.4219	0.0162	0.0689
ADMA	*Prevotella tannerae*	0.3859	0.0292	0.0689
ADMA	*Lactobacillus coleohominis*	0.3617	0.0420	0.0689
**Bacterial translocation**				
BPI	*Lactobacillus helveticus*	0.3642	0.0405	0.0689
SCD14 ^d^	*Lactobacillus vaginalis*	−0.4307	0.0139	0.0689
sCD14	*Streptococcus sobrinus*	−0.3978	0.0242	0.0689
sCD14	*Lactobacillus salivarius*	−0.3584	0.0440	0.0689

^a^, signal joint/beta T-cell receptor excision circles ratio; ^b^, bactericidal permeability increasing protein; ^c^, asymetric dymethilarginine; ^d^, soluble CD14.

## Supplementary Materials

The following are available online at https://www.mdpi.com/2072-6643/11/6/1346/s1, Figure S1: Study profile; Figure S2: Linear discriminative analysis (LDA) effect size (LEfSe) at OTU level between (a) four groups: control, IR, INR, VU; (b) HIV− and HIV-infected groups at baseline. LDA scores for the significant taxa in HIV-infected group are represented on the positive scale (green) and LDA-negative scores represent enriched taxa in control group (red); Figure S3: (a) Principal coordinate analysis (PCoA) based on weighted Unifrac distances between F1.Control, F1.VU, and F2.VU groups. (b) Principal coordinate analysis (PCoA) based on weighted Unifrac distances between F1.Control, F1.IR, and F2.IR groups. (c) Principal coordinate analysis (PCoA) based on weighted Unifrac distances between F1.Control, F1.INR, and F2.INR groups; Figure S4: Principal coordinate analysis (PCoA) based on weighted Unifrac distances between saliva and feces microbiota; Figure S5: Heatmap based on the percentage of taxa that co-occurred (a) at baseline, (b) after prebiotics. We only considered the taxa that presented a percentage, in all the groups, higher than 5%. INR, immunological ART non-responders; IR, immunological ART responders; VU, viremic untreated; Table S1: Statistics of co-occurrence events at baseline and after prebiotic intervention; Table S2: Sample size determination (n1) for groups before prebiotic intervention; Table S3: Sample size determination (n2) for groups before prebiotic intervention; Table S4: Sample size determination (n1) for groups after prebiotic intervention; Table S5. Sample size determination (n2) for groups after prebiotic intervention.
