# Supplementary material for: Modulation of Saliva Microbiota through Prebiotic Intervention in HIV-Infected Individuals
Source: Nutrients. 2019 Jun 14;11(6):1346. doi: 10.3390/nu11061346 (PMC6627446; doi:10.3390/nu11061346)

Figure S2: Linear discriminative analysis (LDA) effect size (LEfSe) at OTU level between (a) four groups: control, IR, INR, VU. (b) HIV- and HIV-infected groups at baseline. LDA scores for the significant taxa in HIV-infected group are represented on the positive scale (green) and LDA-negative scores represented enriched taxa in control group (red).

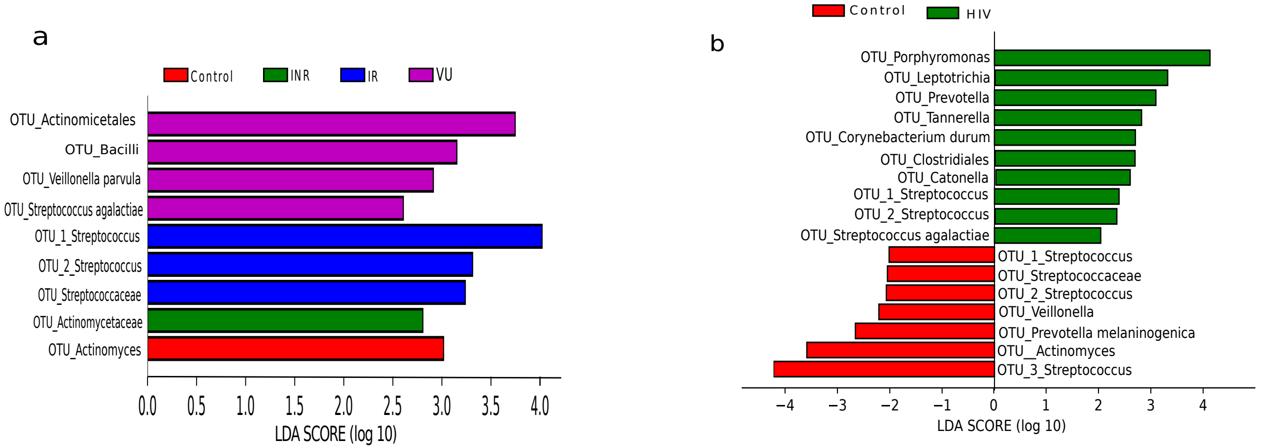

Supplement: Supplementary file 1 [file nutrients-11-01346-s001.zip › FigureS2.pdf]
