# Supplementary material for: Modulation of Saliva Microbiota through Prebiotic Intervention in HIV-Infected Individuals
Source: Nutrients. 2019 Jun 14;11(6):1346. doi: 10.3390/nu11061346 (PMC6627446; doi:10.3390/nu11061346)

Figure S3: (a) Principal Coordinates analysis (PCoA) based on weighted Unifrac distances betweenF1.Control, F1.VU and F2.VU groups. (b) Principal Coordinates analysis (PCoA) based on weighted Unifrac distances between F1.Control, F1.IR and F2.IR groups. (c) Principal Coordinates analysis (PCoA) based on weighted Unifrac distances between F1.Control, F1.INR and F2.INR groups.

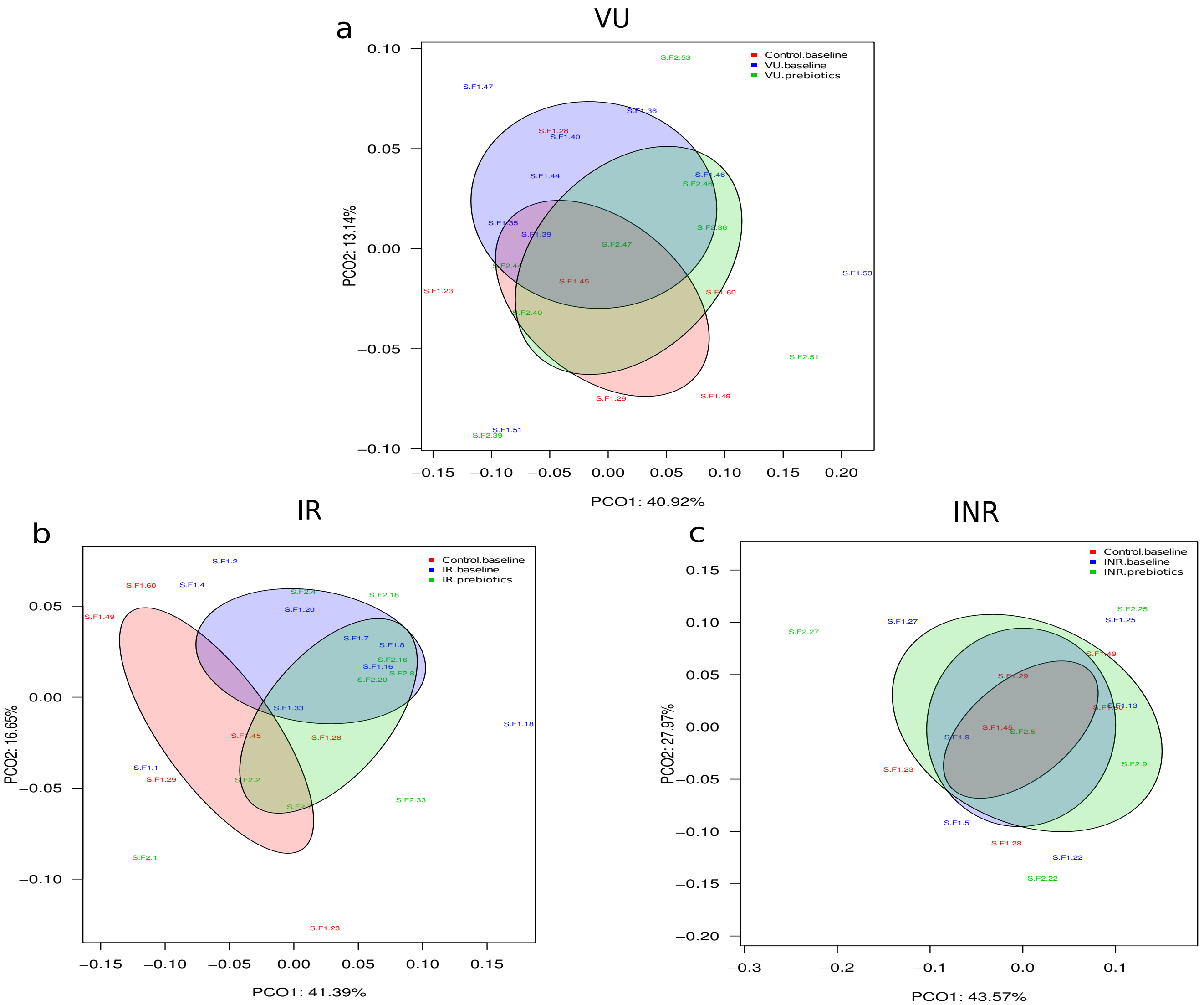

Supplement: Supplementary file 1 [file nutrients-11-01346-s001.zip › FigureS3.pdf]
