# Supplementary material for: Modulation of Saliva Microbiota through Prebiotic Intervention in HIV-Infected Individuals
Source: Nutrients. 2019 Jun 14;11(6):1346. doi: 10.3390/nu11061346 (PMC6627446; doi:10.3390/nu11061346)

Figure S4. Principal Coordinate sanalysis (PCoA) based on weighted Unifrac distances between saliva and feces microbiota.

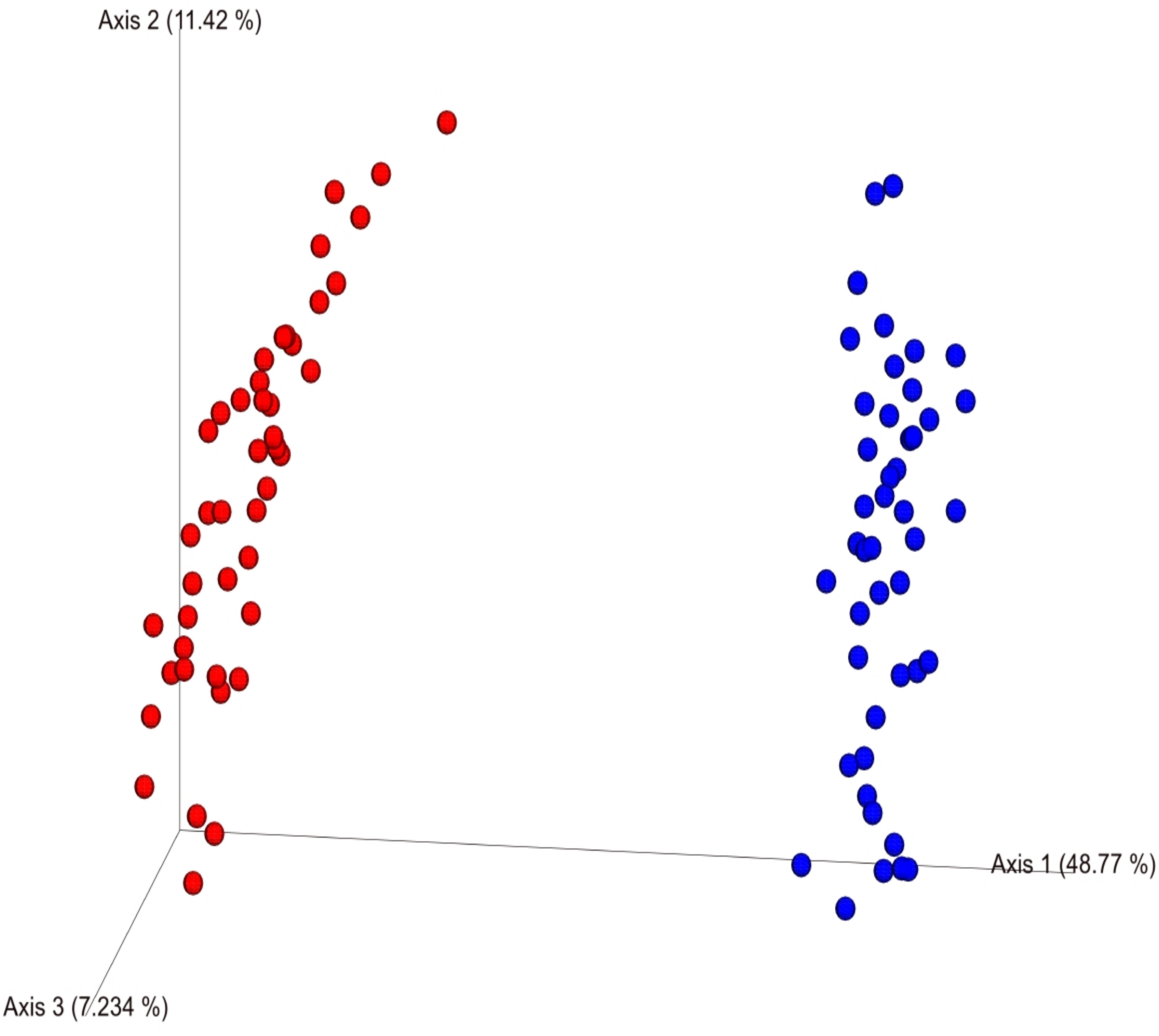

Supplement: Supplementary file 1 [file nutrients-11-01346-s001.zip › FigureS4.pdf]
