# Supplementary material for: Modulation of Saliva Microbiota through Prebiotic Intervention in HIV-Infected Individuals
Source: Nutrients. 2019 Jun 14;11(6):1346. doi: 10.3390/nu11061346 (PMC6627446; doi:10.3390/nu11061346)

Figure S5. Heatmap based on the percentage of taxa that co-occurred. (a) At baseline (b) After prebiotics . We only considered the taxa that presented a percentage, in all the groups, higher than 5%. INR, immunological ART non-responders; IR, immunological ART responders; VU, viremic untreated

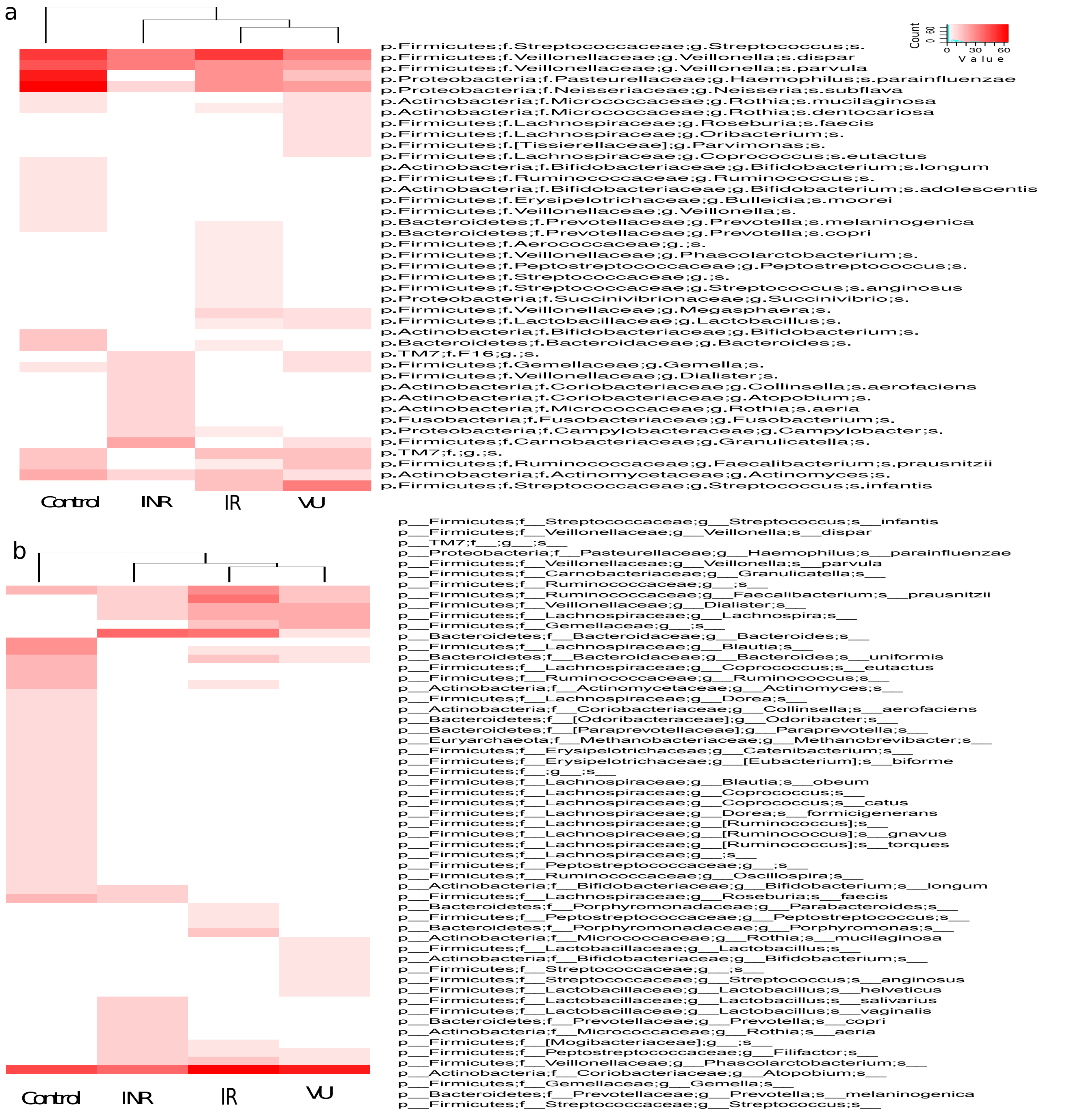

Supplement: Supplementary file 1 [file nutrients-11-01346-s001.zip › FigureS5.pdf]
