# Supplementary figures and images for: Modulation of Saliva Microbiota through Prebiotic Intervention in HIV-Infected Individuals
Source: Nutrients. 2019 Jun 14;11(6):1346. doi: 10.3390/nu11061346 (PMC6627446; doi:10.3390/nu11061346)

**Figure S1.** Study profile

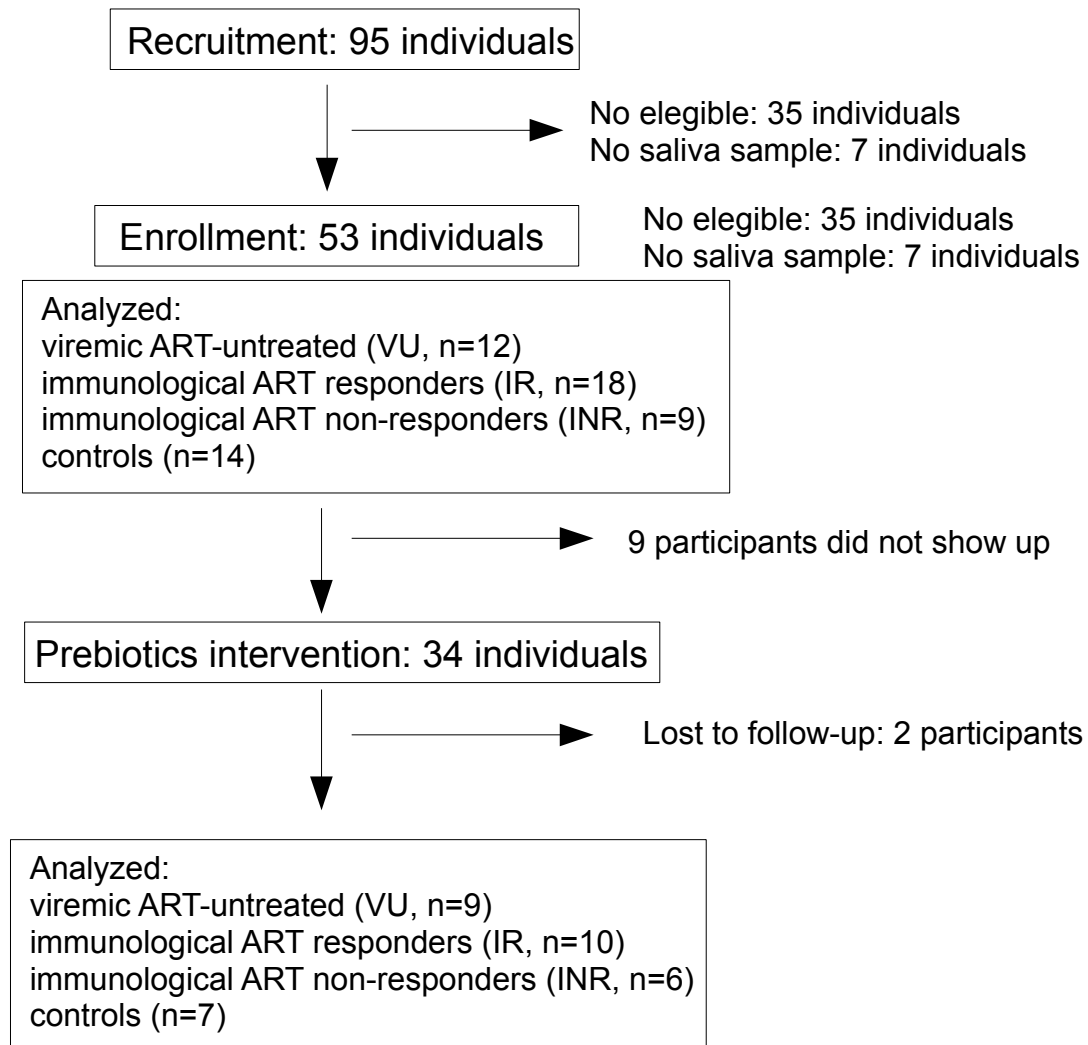

Supplement: Supplementary file 1 [file nutrients-11-01346-s001.zip › FigureS1.pdf]
