# Supplementary material for: Modulation of Saliva Microbiota through Prebiotic Intervention in HIV-Infected Individuals
Source: Nutrients. 2019 Jun 14;11(6):1346. doi: 10.3390/nu11061346 (PMC6627446; doi:10.3390/nu11061346)
Supplement: Supplementary file 1 [file nutrients-11-01346-s001.zip › Table S1.doc]

Table S1. Statistics of co-occurrence events at baseline and after prebiotic intervention

a, baseline

b, after prebiotics

INR, immunological ART non-responders; IR, immunological ART responders; VU, viremic untreated.
